# Bioclinical Parameters Driving Decision-Making of Subsequent Lines of Treatment in Metastatic Castration-Resistant Prostate Cancer

**DOI:** 10.1155/2014/909623

**Published:** 2014-05-28

**Authors:** A. Irelli, G. Bruera, K. Cannita, E. Palluzzi, G. L. Gravina, C. Festuccia, C. Ficorella, E. Ricevuto

**Affiliations:** ^1^Medical Oncology, S. Salvatore Hospital, University of L'Aquila, 67100 L'Aquila, Italy; ^2^Department of Biotechnological and Applied Clinical Sciences, University of L'Aquila, 67100 L'Aquila, Italy; ^3^Radiotherapy, S. Salvatore Hospital, University of L'Aquila, 67100 L'Aquila, Italy

## Abstract

Different options are available as second-line treatment of metastatic castrate-resistant prostate cancer: cabazitaxel, abiraterone, and enzalutamide. Phase III studies evaluating cabazitaxel and the two hormonal agents have been shown to significantly prolong overall survival compared to mitoxantrone and placebo, respectively. Several studies have also demonstrated feasibility and activity of docetaxel rechallenge in case of a sufficient progression-free interval (3–6 months), good performance status, and previous acceptable safety profile, thus providing an additional treatment option in clinical practice. Clinical and biological parameters should be considered to tailor II line treatment. In clinical practice, we can primarily evaluate patients' fitness according to age, performance status, symptomatic disease, comorbidities, and expected safety profile of each drug. Different prognostic/predictive factors may be considered, such as presence of bone-limited or visceral metastases, length of androgen deprivation therapy (ADT) before chemotherapy, time to progression after docetaxel, Gleason score, PSA doubling time, and serum testosterone, even if their clinical relevance is still debated. This review will discuss current options of innovative drugs sequencing and selection according to bioclinical parameters.

## 1. Treatment Options in Clinical Practice after Progression to Docetaxel

In clinical practice, different treatment options are now available after progression to docetaxel in the management of castrate-resistant prostate cancer (CRPC) patients: cabazitaxel, abiraterone and enzalutamide, and other drugs are currently under evaluation for clinical recommendation, such as alpha emitter radium-223 [1–7].

### 1.1. Cabazitaxel

Cabazitaxel is a novel taxane that inhibits microtubule depolymerization and cell division by binding tubulin, resulting in cell cycle arrest. It showed antitumor activity in models resistant to docetaxel and is able to cross the blood-brain barrier [1].

In the phase III randomized Tropic trial, patients progressing during or after docetaxel were randomized to cabazitaxel 25 mg/m² or mitoxantrone 12 mg/m². At median follow-up 12.8 months, overall survival was 15.1 versus 12.7 months (HR 0.70, *P* < 0.0001) [1]. Treatment with cabazitaxel was prognostic for survival ≥2 years, 27% versus 16%, respectively [2]. Median progression-free survival was 2.8 months compared to 1.4 months (HR 0.74, *P* < 0.0001), with a significantly higher tumor response rate 14.4% compared with 4.4% (*P* = 0.0005) with cabazitaxel and mitoxantrone, respectively. More, median time to PSA progression was 6.4 months compared to 3.1 months, *P* = 0.0010; PSA response rate was 39.2% compared to 17.8%, *P* = 0.0002. No statistical differences were reported in progression of pain and response to pain. Grade 3-4 related adverse events significantly different in the cabazitaxel and mitoxantrone arms were: neutropenia (82% versus 58%, febrile 8% versus 1%) and diarrhea (6% versus <1%). Thus, prophylactic use of granulocyte colony-stimulating growth factors are strongly recommended, and a lower dose of cabazitaxel (e.g. 20 mg/m^2^) may be considered and is now under evaluation [1].

Overall survival benefit was reported in patients who received a cumulative dose of docetaxel <225 mg/m^2^, who progressed during docetaxel (29%) and who progressed during and within 3 months after docetaxel (45%). The survival advantage of cabazitaxel also persisted in patients with measurable disease or pain, most pronounced in patients with performance status 0-1, and disease progression within <3 months from docetaxel. PSA immediate reduction or flare were associated with a OS benefit.

### 1.2. Abiraterone

Abiraterone is a selective inhibitor of cytochrome P450, CYPC17, and inhibits the residual amount of androgenic steroid predominantly produced in adrenal gland [3].

In phase III, randomized COU-AA-301 trial, patients were randomized to receive 1000 mg of abiraterone acetate or placebo, with 5 mg of prednisone twice daily. At median follow-up 12.8 months, abiraterone significantly increased overall survival, 14.8 versus 10.9 months, *P* < 0.001; and also time to PSA progression (10.2 versus 6.6 months, *P* < 0.001), progression-free survival (5.6 months versus 3.6 months, *P* < 0.001), and PSA response rate (29% versus 6%, *P* < 0.001). An attenuation of pain was significantly higher with abiraterone (44% versus 27%, *P* = 0.002), with progression of pain at 6 (22% versus 28%), 12 (30% versus 38%) and 18 months (35% versus 46%), and time until progression of pain was 7.4 months compared with 4.7 months, respectively [3].

Abiraterone produced similar improvement in median overall survival in patients with (4.6 months) and without (4.8 months) visceral disease, hazard ratios 0.79 and 0.69 (*P* = 0.0001), respectively; HRs for rPFS were 0.60 (*P* = 0.0002) and 0.68 (*P* < 0.0001); PSA response rates were 28% versus 7% and 30% versus 5% (both *P* = 0.0001), respectively; ORRs were 11% versus 0% (*P* = 0.0058) and 19% versus 5% (*P* = 0.0010), respectively [4].

A lower percentage of skeletal related events were reported with abiraterone at 6 months (18% versus 28%), 12 months (30% versus 40%), and 18 months (35% versus 40%), and the time at the first skeletal event was 9.9 months compared to 4.9 months. More common adverse events were hypokalemia, fluid retention, and hypertension, largely abrogated by low-dose glucocorticoids [3].

At an update follow-up 20.2 months, significantly increased clinical outcomes were confirmed in abiraterone arm: overall survival (15.8 versus 11.2 months, *P* < 0.0001), median time to PSA progression (8.5 versus 6.6 months, *P* < 0.0001), radiologic progression-free survival (5.6 versus 3.6 months, *P* < 0.0001), and PSA response rate (29.5% versus 5.5%; *P* < 0.0001) [5].

### 1.3. Enzalutamide

Enzalutamide is an androgen-receptor-signaling inhibitor that inhibits nuclear translocation of the androgen receptor, DNA binding, and coactivator recruitment. It also has a greater affinity for the receptor and has no known agonistic effects [6].

In the AFFIRM phase 3 randomized trial, at median follow-up 14.4 months, enzalutamide 160 mg significantly increased clinical outcome compared to placebo: median overall survival 18.4 and 13.6 months, with 37% reduction in the risk of death (*P* < 0.001); radiographic progression-free survival 8.3 and 2.9 months (*P* < 0.001); median time to PSA progression 8.3 and 3.0 months (*P* < 0.001); median time to first skeletal event 16.7 and 13.3 months (*P* < 0.001). More frequent adverse events were fatigue, diarrhea, hot flashes, musculoskeletal pain and headache [6].

### 1.4. Radium-223

Radium-223 dichloride (radium-223), an alpha emitter, selectively targets bone metastases with alpha particles. Alpha emitter radium-223 recently showed a significantly improved overall survival (14.9 versus 11.3 months, *P* < 0.001) in the phase III ALSYMPCA study [7].

## 2. Bioclinical Parameters to Tailor Treatment Strategies

### 2.1. Patients' Fitness

Prostate cancer is prevalently an elderly disease: median age at diagnosis 67 years [8]. Patient's distribution according to age is: young < 65 years, elderly ≥ 65 years, and specifically young-elderly (65–74 years), old-elderly (75–84 years), and oldest (≥85 years).

In the decision-making process different clinical parameters, such as age, functional, nutritional, and comorbidity status should be considered. Different geriatric assessment evaluations can be used to properly define patients' fitness and select tailored treatment options, with different schedules and drug dosages. In our clinical practice experience, Cumulative Illness Rating Scale (CIRS) is commonly used to objectively define comorbidity status in the individual patient [9]. CIRS evaluates the presence and the severity (absent, slight, moderate, severe, and very serious) of the coexisting disorders in the major organs (heart, vascular, respiratory, eye, ear nose and throat, upper digestive tract, lower digestive tract, liver, kidney, genitourinary, musculoskeletal, neurological, and endocrinological disorders). More, functional status is evaluated using instrumental activities of daily living measured by ADL [10] and IADL [11]. Patients are classified into different CIRS stages: primary, if absent comorbidities and functional independent; intermediate, <3 mild and/or moderate categories, and dependent IADL; secondary, ≥3 categories or severe comorbidity, and dependent ADL. In this decision-making process, young and young-elderly patients with primary and/or intermediate CIRS stage can be considered fit for standard treatments, while old-elderly and/or secondary CIRS stage patients are not eligible for conventional treatments and should be considered for treatment modifications (dose, schedule), to properly balance safety and efficacy.

Moreover, nutritional status can be estimated by the variation of weight during the previous 3 months: good nutritional status, weight loss <5%, risk of malnutrition, 5–10%, severe malnutrition, >10%. All these clinical features should be considered to define fitness and discriminate patients fit or unfit for specific drugs (Table 1).

#### 2.1.1. Age

In the interim analysis of the European compassionate use program, cabazitaxel-related adverse events were manageable in clinical practice in elderly population (≥70 years). Dose intensity, dose delays and dose reductions were similar in ≥70 years and younger patients. Diarrhea was the most common adverse event, usually mild with grade ≥3 in 2.2%. Prophylactic G-CSF was more commonly administered in patients ≥70 years [12]. However, haematological toxicity of cabazitaxel appeared similar to younger patients. In multivariate analysis, advanced age (≥75 years), first cabazitaxel cycle and low neutrophil count before cabazitaxel (<4000/mm^3^) were associated with an increased risk of grade ≥3 neutropenia and/or neutropenic complications [13]. In the cabazitaxel compassionate-use and early access programs, prevalently enrolling ≤75 years patients (81.6%), adverse events were more frequent in older patients (64.2% versus 54.8%), resulting in treatment discontinuation in 24.4% and 36.4%, respectively. Grade ≥3 neutropenia was observed in 25.8% and 17.0% of < and ≥75 years patients, respectively [14].

In the post hoc analysis of COU-AA-301, grade 3/4 adverse events with abiraterone occurred in 62% and 60% of elderly (≥75 years) and younger (<75 years) patients, respectively. Incidences of hypertension and hypokalemia were similar in both age subgroups [15].

In the AFFIRM trial, similar safety profiles were observed in elderly and younger patients [16] (Table 2).

#### 2.1.2. Performance Status

About 20% prostate cancer patients show ECOG PS 2 at diagnosis. Among overall PS 2 population who progressed after docetaxel, experimental treatments decreased risk of death by 26% (*P* = 0.035); activity was similar in PS 0 or 1 with reduced risk of death of 31%. The significant reduction of risk of death was confirmed for hormonal therapies, abiraterone and enzalutamide (HR = 0.72; *P* = 0.046), but not for chemotherapy (HR = 0.81, *P* = 0.43) [17] (Table 3).

### 2.2. Symptomatic Disease

Almost 70% metastatic PC patients have a high incidence of bone lesions that correlate with skeletal-related events (SREs), defined as fracture (pathological vertebral and/or nonvertebral), bone radiation, bone surgery, and spinal cord compression, including or not hypercalcemia [18]. SRE rate can depend on: number of bone metastases, metastatic site, type of lesion, treatment especially preventive one. Several studies have attempted to correlate skeletal involvement with survival. Staging systems based on scintigraphic distribution (axial versus appendicular) or number of metastases showed a significant association with survival. Median survivals of patients with low, intermediate, or extensive skeletal involvement evaluated by the bone scan index, based on the percentage of tumor involvement multiplied by the weights of each of the 158 bones, were 18.3, 15.8, and 8.1 months, respectively [19]. SREs do not include pain, although persistent prostate cancer-induced bone pain (PCIBP) is one of the most distressing symptoms [20]. In clinical practice, treatment choice for metastatic CRPC patients is driven primarily by asymptomatic or minimally symptomatic, versus symptomatic disease [21].

## 3. Prognostic and Predictive Factors

Prognostic factor is any clinical and/or biological parameter that correlates with clinical outcome and that can therefore be used in clinical practice in the decision-making process; instead, predictive factor is any clinical or biological parameter that significantly correlate with activity and efficacy of a specific drug, and that can be used to select specific population suitable for a specific therapy or targeted agent. However, more frequently, most parameters show a prognostic and predictive relevance. In metastatic CRPC, prognostic factors usually evaluated in clinical trials are symptomatic disease, presence of visceral metastases, short duration of hormone therapy before chemotherapy (16–20 months), and baseline levels of serum testosterone. Predictive factors are disease progression during or <3 months after docetaxel, Gleason score, and PSA doubling time.

### 3.1. Metastatic Sites

All phase 2 or 3 clinical trials showed a significant trend over time (*P* = 0.001) of increasing proportions of patients with non-osseous metastasis (lymph node, visceral, and soft tissue) (1.4% per year increase), with stable proportion of liver involvement [22]. The presence of visceral metastases is a negative prognostic factor and may confer less sensitivity to hormone therapy. In an updated analysis of COU-AA-301 trial, overall survival benefit of abiraterone was similarly improved in patients with visceral (liver or lung, but not nodal-only metastases) (4.6 months) and without visceral metastases (4.8 months), even if not significantly for visceral disease. Abiraterone conferred significant 40% and 32% reductions in the risk of radiographic progression or death in patients with or without visceral disease, respectively. Soft-tissue objective response rates were superior with abiraterone in both groups. PSA response rates were significantly improved by abiraterone in both groups [23].

### 3.2. Previous Duration of Hormonal Therapy

In the Institut Gustave Roussy clinical trials database, median duration of prostate cancer sensitivity to androgen deprivation therapy ≥16 months significantly and strongly predicted for both PSA response (58% versus 18%, *P* = 0.01) and PFS (5 and 3 months, *P* < 0.043), in >16 months and <16 months subgroups, respectively. So, it may represent a significant predictive factor for efficacy of subsequent endocrine manipulations [24].

### 3.3. Disease Progression during or <3 Months after Docetaxel

None of docetaxel refractory patients had PSA, radiological, or clinical response to abiraterone. In 16% metastatic CRPC patients treated with docetaxel followed by abiraterone PSA decline ≥50% was observed. There was no relationship between length of time on LHRH agonist and PSA response to abiraterone [25].

### 3.4. PSA Doubling Time

In a cohort of men with biochemical recurrence after prostatectomy, at a median follow-up 4 years, 29.3% developed metastases, and there was a statistically significant inverse correlation between PSA doubling time and PSA at metastasis (*P* = 0.02), but not for Gleason score [26].

### 3.5. Testosterone Serum Level

In 9 first line phase II-III trials, testosterone under castration level (<50 ng/dL) was prognostic for overall survival, and PSA response to salvage hormone-therapy differed depending on testosterone serum levels: median overall survival was 22.4 months compared to 32.7 months in patients with testosterone level under and higher median value (11.53 ng/dL) (*P* = 0.0162), and PSA response 21.74% and 55.6%, respectively [27]. In clinical practice, serum testosterone <20 ng/dL seems to represent an adequate castration level [28].

### 3.6. Gleason Score

Initial Gleason score between 8 and 10 may be predictive factor of a poor response to hormonal treatment including abiraterone [29].

## 4. Clinical Algorithm to Define the Sequence of Administration in Second and Subsequent Lines 

After docetaxel, treatment options for metastatic CRPC include cabazitaxel, abiraterone acetate, and enzalutamide. Phase III studies evaluating cabazitaxel and abiraterone included patients with similar baseline features: median age 68 and 69 years, 18% and 28% patients > 75 years, PSA basal level 143.9 and 128.8 ng/mL, 46% and 44% symptomatic disease, respectively. In the TROPIC trial, 6% patients were treated for >2 lines. All outcomes were significantly increased in the experimental arms: median overall survival 15.1 and 14.8 months, with HR for risk of death 0.70 and 0.65, compared to mitoxantrone and placebo, in the TROPIC and COU-AA-301 trials, respectively. Thus, in second-line setting, overall survival was equivalently improved by cabazitaxel and abiraterone, even if survival of control arms were slightly different, 10.9 versus 12.7 months for prednisone and mitoxantrone, respectively (Table 4).

Optimal sequence of cabazitaxel and abiraterone after docetaxel was evaluated in 130 patients, significantly more frequently (*P* < 0.001) treated with docetaxel→cabazitaxel→abiraterone (67.7%) compared with docetaxel→abiraterone→cabazitaxel (32.3%). The sequence docetaxel-abiraterone was prevalent among patients not eligible to receive more than 2 lines of treatment, while the sequence docetaxel-cabazitaxel was preferable for patients suitable for 3 lines of treatment. Thus, the unbalance favoring the sequence docetaxel-cabazitaxel-abiraterone could be partially related to the clinical* a priori* selection [30]. A retrospective analysis was conducted on an American electronic database (iKnowMed Electronic Health Records). A total of 113 patients were treated with sequential docetaxel, cabazitaxel, and abiraterone: 77 with the sequence docetaxel-cabazitaxel-abiraterone and 36 with docetaxel-abiraterone-cabazitaxel. Overall survival was superior in the sequence docetaxel-cabazitaxel-abiraterone versus docetaxel-abiraterone-cabazitaxel, 18.2 versus 11.8 months, respectively (HR = 0,12) [31]. Cabazitaxel may be considered an option for patients refractory to first-line docetaxel, with very early development of CRPC, visceral metastases and a very short PSA doubling time.

In the compassionate use of abiraterone after docetaxel, 18.4% subsequently received cabazitaxel, prevalently at 20 mg/m^2^, and obtained 26% PSA control. Median survival from docetaxel was 32.0 months, from abiraterone 16.1 months, from cabazitaxel 8.2 months. Non progression to docetaxel was associated with longer survival with cabazitaxel, 43.1 versus 17.4 months (*P* = 0.049), while not to abiraterone [32]. Among patients treated with third-line cabazitaxel after abiraterone, 20% had a partial response [33].

In metastatic CRPC patients previously treated with abiraterone, docetaxel rechallenge resulted in PSA decline ≥50% in 26%, with partial responses 11%, time to PSA progression 4.6 months, and overall survival 12.5 months. No responses to docetaxel were observed in abiraterone-refractory patients. These data may support the hypothesis of cross-resistance between these agents and preclinical evidence that docetaxel antitumour activity may be related to its impact on androgen receptor signaling. The high intratumoral androgens in patients discontinuing abiraterone could reduce docetaxel antitumour activity, and androgen receptor overexpression or mutation may also contribute to docetaxel resistance. More, abiraterone activity seems to be different according to its sequencing with docetaxel [34].

PSA decrease ≥50% was reached in 41.3% patients treated with cabazitaxel, with median overall survival 13.3 months and median clinical and/or radiological progression-free survival 6.5 months. Abiraterone or enzalutamide were given before and after cabazitaxel in 33% and 16% patients, respectively. In patients treated with abiraterone or enzalutamide after and before cabazitaxel, median overall survival from docetaxel was 65 months versus 39 months, respectively. Overall survival was significantly reduced in patients with ECOG 2, alkaline phosphatase ≥1.5 upper limit of normal, lymph node involvement, and significantly prolonged in patients treated with ≥2 docetaxel lines, prior curative therapy, PSA decrease ≥30% with cabazitaxel and in patients treated with abiraterone/enzalutamide after cabazitaxel. Thus, patients treated with new hormonal agents after cabazitaxel seemed to experience a prolonged overall survival [35].

After failure of abiraterone, enzalutamide achieved response rate 2.9%, time to progression 4.0 months: PSA decline >50% in 45.7%, specifically 43.8% and 15.8% in abiraterone-sensitive and abiraterone-insensitive patients, respectively. 48.6% of patients were enzalutamide-resistant and showed a rising PSA as the best response [36].

In the enzalutamide expanded access program after progression to docetaxel and abiraterone, 39% of patients showed >50% PSA reduction, 60% and 23% in patients sensitive and insensitive to abiraterone, respectively [37].

In an indirect comparative effectiveness analysis between enzalutamide and abiraterone using the results of AFFIRM and COU-AA-301 trials, respectively, based on the assumption that the relative effects of each drug compared to placebo were the same, enzalutamide appears to be more effective than abiraterone in terms of time to PSA progression, radiologic progression-free survival and PSA response, and overall survival was not different: hazard ratios for overall survival were 0.63 (0.53–0.75) and 0.66 (0.56–0.79); indirect estimate for enzalutamide versus abiraterone 0.96 (0.75–1.22); HR for time to PSA progression 0.40 (0.35–0.47) and 0.67 (0.59–0.78); indirect estimate of HR for enzalutamide versus abiraterone 0.60 (0.49–0.74); odds ratios for PSA response 77.08 (34.02–174.70) and 5.49 (3.84–7.84); indirect estimate for enzalutamide versus abiraterone 14.04 [38].

Several studies have demonstrated feasibility and activity of docetaxel rechallenge in metastatic CRPC patients in case of a sufficient progression-free interval (3–6 months), good ECOG PS, and previous acceptable safety profile, thus providing an additional treatment option in clinical practice: overall biochemical response rate (PSA reduction > 50%) 66%, overall survival 32 months with projected 2-year overall survival from first docetaxel administration 77.5%. Multivariate analysis showed that time slope-log PSA, time from the previous cycle, and response to the previous cycle were predictive of response to rechallenge [39].

Thus, in the complex management of metastatic CRPC different clinical and biological parameters should be considered in clinical practice to better define patients' fitness and select treatment strategy to optimize clinical outcome.

## Figures and Tables

**Table 1:** Patients' features.

Clinical trial	Median age	Range	≥75 years	PS 2	Visceral metastases
TROPIC	68	62–73	18%	7%	25%
COU-AA-301	69	42–95	28%	10%	11% (liver metastases)
AFFIRM	69	41–92	25%	8.5%	23.2%

**Table:** (a) Cabazitaxel

	<70 years	≥70 years
Diarrhea	3.3%	2.2%
Fatigue	4%	4.3%
Asthenia	1.4%	4.9%
Neutropenia	15%	19.7%
Febrile neutropenia	5.2%	5.5%
Anemia	5%	4.3%

**Table:** (b) Enzalutamide

	≥75 years	<75 years
Fatigue	40%	32%
Nausea	32%	33%

**Table 3:** Overall survival.

Experimental treatments	Control treatment	HR	95% CI	*P* value
Cabazitaxel	Mitoxantrone	0.81	0.48–1.37	0.43
Abiraterone and enzalutamide	Placebo	0.72	0.52–0.99	0.046
All treatments in PS 2 patients		0.74	0.56–0.98	0.035

**Table 4:** Clinical outcome.

	Cabazitaxel		Mitoxantrone	
OS (months)	15.1		12.7	*P* < 0.0001
Median followup (months)	12.8			HR 0.70 (0.59–0.83)
PFS (months)	2.8		1.4	*P* < 0.0001
PSA progression (months)	6.4		3.1	*P* = 0.001
PSA response rate (%)	39.2		17.8	*P* = 0.0002
ORR (%)	14.4		4.4	*P* = 0.0005

	Abiraterone		Placebo	

OS (months)	14.8		10.9	*P* < 0.001
Median followup (months)	12.8			HR 0.65 (0.54–0.77)
PFS (months)	5.6		3.6	*P* < 0.001
PSA progression (months)	10.2		6.6	*P* < 0.001
PSA response rate (%)	29		6	*P* < 0.001
ORR (%)	14		2.8	*P* < 0.001

	Enzalutamide		Placebo	

OS (months)	18.4		13.6	*P* < 0.001
Median followup (months)	14.4			HR 0.63 (0.53–0.75)
PFS (months)	8.3		2.9	*P* < 0.001
PSA progression (months)	8.3		3	*P* < 0.001
PSA response rate (%)	54		2	*P* < 0.001
ORR (%)	29		4	*P* < 0.001
